# Comparative evaluation of salivary glands proteomes from wild *Phlebotomus papatasi* *–*proven vector of zoonotic cutaneous leishmaniasis in Iran

**DOI:** 10.1002/vms3.368

**Published:** 2020-09-24

**Authors:** Seyedeh Maryam Ghafari, Sahar Ebrahimi, Mahmoud Nateghi Rostami, Ali Bordbar, Parviz Parvizi

**Affiliations:** ^1^ Molecular Systematics Laboratory Parasitology Department Pasteur Institute of Iran Tehran Iran; ^2^ Parasitology Department Pasteur Institute of Iran Tehran Iran

**Keywords:** HPLC, *Phlebotomus papatasi*, PpSP15, Salivary glands, Zoonotic Cutaneous Leishmaniasis

## Abstract

**Background:**

Zoonotic Cutaneous Leishmaniasis is increasing in the world and *Phlebotomus papatasi* as a proven vector was considered in different aspects for disease control. Sandfly saliva contains proteins which provoke host immune system. These proteins are candidates for developing vaccines.

**Objectives:**

The main purpose of this research was comparing evaluation of salivary glands proteomes from wild *P. papatasi*. Extracting these proteins and purifying of original SP15 as inducer agent in vector salivary glands from endemic leishmaniasis foci were other objectives.

**Methods:**

Adult sandflies were sampled using aspirators and funnel traps from three endemic foci in 2017–2018. Each pair of salivary glands of unfed females was dissected and proteins were extracted using thermal shocking and sonication methods. Purification was performed through RP‐HPLC. All equivalent fractions were added together in order to reach sufficient protein concentration. Protein content and profile determination were examined with SDS‐PAGE.

**Results:**

The protein concentration of whole‐salivary glands of specimens was determined approximately 1.6 µg/µl (Isfahan) and 1 µg/µl (Varamin and Kashan). SDS‐PAGE revealed 10 distinct bands between 10 and 63 kDa. Analysis of proteomes showed some similarities and differences in the chromatograms of different foci. SDS‐PAGE of all collected fractions revealed SP15‐like proteins were isolated in 24 min from Varamin, 26 to 30 min from Kashan and 29.4 min from Isfahan and were around 15 kDa.

**Conclusions:**

Isolation of salivary components of Iranian wild *P. papatasi* is very important for finding potential proteins in vaccine development and measuring control strategy of zoonotic cutaneous leishmaniasis in Iran and this could be concluded elsewhere in the world.

## Graphical abstract

Wild *Phlebotomus papatasi* salivary glands proteomes, which provoke host immune system, were compared and evaluated. The protein concentration of whole‐salivary glands of specimens was determined for measuring control strategy of zoonotic cutaneous leishmaniasis

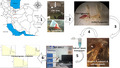

## 1 INTRODUCTION

*Phlebotomus papatasi* (Scopoli, 1786) as only well‐known proven vector of zoonotic cutaneous leishmaniasis (ZCL) in Iran has been under a lot of considerations in different aspects of researches (Killick‐Kendrick, 1990; Parvizi & Ready, 2008). In Old World foci of ZCL, the blood‐feeding females of *P. papatasi* (Diptera: Psychodidae: Phlebotominae) sandfly are incriminated to be the natural vector of protozoan *Leishmania major*, the causative agent of the neglected tropical disease ZCL. In Iran and many countries, ZCL is an endemic disease and is increasing in many foci. ZCL originally is a disease of gerbils. Sandflies, which transmit *Leishmania* parasites, live and breed in gerbil burrows. The function of *P. papatasi* salivary proteins is provoke of human immune system in ZCL foci (Mahamdallie & Ready, 2012; Ready, 2013; Zahirnia et al., 2018). Few researches are available on Iranian wild *P. papatasi* salivary glands proteins. Biological and environmental conditions were studied previously about affecting on protein content of salivary glands (Hosseini‐Vasoukolaei, Mahmoudi, et al., 2016).

Differential expression of salivary protein genes was described under various physiological conditions (Hosseini‐Vasoukolaei, Idali, et al., 2016). There is not any report on purification and characterization of salivary glands proteins in *P. papatasi* from Iranian foci. The most salivary glands proteins have been purified and characterized from colonies breeding in insectarium (Marzouki et al., 2012; Valenzuela et al., 2001; Vlkova et al., 2014). In this investigation, wild *P. papatasi* was considered and worked from endemic foci of ZCL in Iran. As we are aware, SP15 family of proteins is the most abundant in the majority of sandfly salivary glands but not in cDNA library (Abdeladhim et al., 2012). These proteins with unknown functions contain PpSP15 from *P. papatasi* saliva (Acc. no. AAL11047) could elicit specific humoral and cellular immunity. PpSP15 is able to protect immunized mice against *Leishmania major* (Oliveira et al., 2008; Valenzuela et al., 2001; Vlkova et al., 2014). SP15 as an antigen in vaccine production could enhance protective efficacy against leishmaniasis (Zahedifard et al., 2014).

Salivary gland proteins from colonized sandflies do not cause protection against infection with *L. major* in the case of pre‐exposure to wild specimens (Ben Hadji Ahmed et al., 2010).

Nevertheless there are many endeavours to vaccine development against leishmaniasis, it has been failed because of some reasons. The origin of utilized SP15 as the inducer agent on human immunity system is important. Almost purification of salivary gland proteins has been performed on reared sandflies rather than wild‐caught sandflies. Extracting salivary gland proteins from wild‐caught *P. papatasi* was one of the purposes of this research. Purifying of SP15 as an adjuvant in vaccine production and comparison between protein profiles from different ZCL foci were other objectives.

## 2 MATERIALS AND METHODS

### 2.1 Sandfly collection, identification, and salivary gland preparation

**FIGURE 1:**
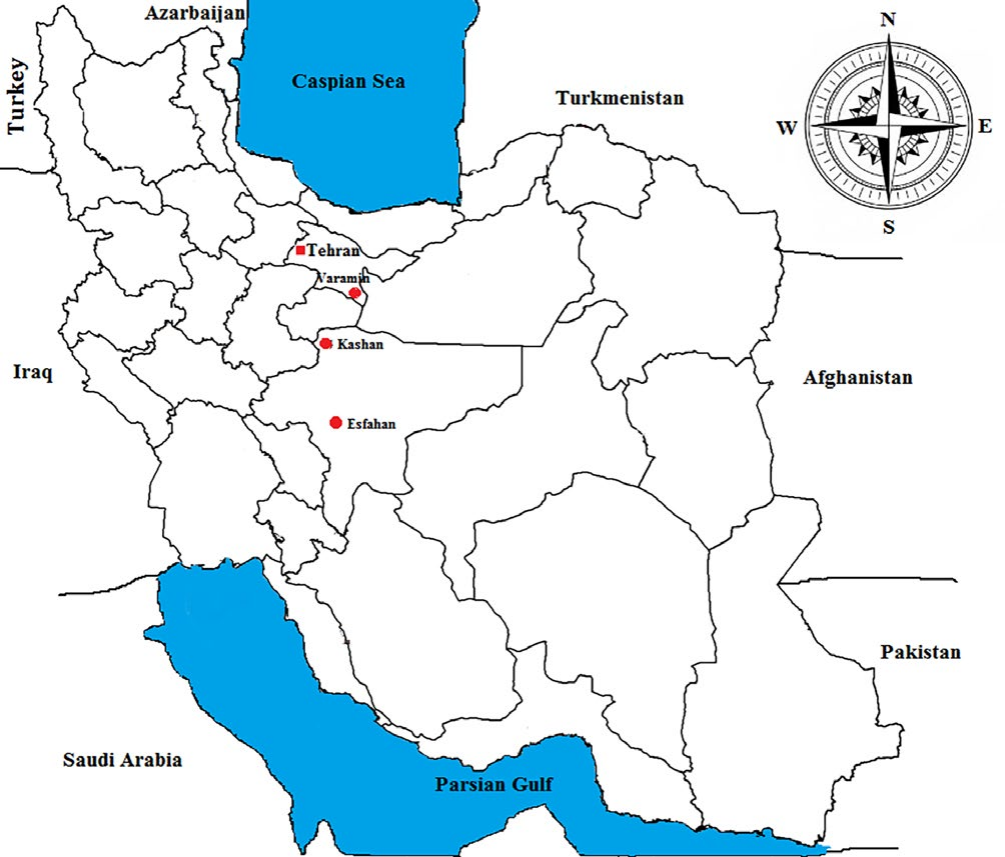
Geographical locations of ZCL foci where sandflies were sampled

Sand flies were collected during the activity season of adult sandflies from three ZCL locations of Iran in 2017 and 2018. Female *P. papatasi* were identified and prepared for protein extraction from salivary glands. The collections were made at the villages near the cities in Isfahan and Tehran provinces, geographic coordinates of these regions were as follows: Kashan (33°58′59.09ʺN, 51°26′11.18ʺE); Isfahan (32°39′8.86ʺN, 51°40′28.63ʺE) and Varamin: (35°19′27″N, 51°38′44″E; Figure 1; https://www.geodatos.net/en/coordinates/iran).

Wild adult sandflies were collected using aspirators and funnel traps from domestic animal shelters and rodent borrows respectively. The collected specimens put in microtubes without any buffer and were frozen (−20°C). All collected specimens were kept on ice and transferred to Pasteur institute of Iran in Tehran and stored in −70°C. All adult sandflies were identified using morphological characters of the head and abdominal terminalia, following a dissection for salivary glands with sterilized forceps and micro‐needles (Parvizi et al., 2003). After dissecting, each pair of salivary glands of unfed females *P. papatasi* was moved into a micro‐tube without any buffer and flash frozen at −70°C for protein extraction.

### 2.2 Protein extraction

Isolated salivary glands of the female *P. papatasi*, encompassed a rapid freeze‐thaw process (−70°C for 2 min then 37°C water bath for 1 min), following sonication on ice for 1 min at 45% and for 5 min at 90% power using an ultrasonic processor (Hielscher; Ben Hadji Ahmed et al., 2010; Hosseini‐Vasoukolaei, Mahmoudi, et al., 2016). Extraction product was centrifuged for 1 min at 14,000 rpm. Supernatant was filter sterilized with centrifugation at 14,000 rpm for 30 min through a 0.22‐µm Ultra‐free centrifugal filter unit (Millipore; Geraci et al., 2014).

For removing undesirable substances, cold acetone (four times, −20°C) was added to protein precipitation. The tube was incubated at −20°C for 60 min. The supernatant containing the interfering substance was decanted and the pellet contains protein stored until usage (Fic et al., 2010; Peach et al., 2015). For every experimental test, the protein pellet was re‐dissolved in phosphate buffer saline (pH = 7) that is compatible with the downstream applications.

### 2.3 Protein concentration determination and SDS‐PAGE

A microplate reader spectrophotometer (Synergy‐HTX, Biotek) fulfilled the protein concentration directly. The concentration of protein was determined in 280 nm.

Sodium dodecyl sulphate‐polyacrylamide gel electrophoresis (SDS‐PAGE) was carried out according to standard method (Laemmli, 1970). The samples were loaded onto a 15% polyacrylamide gel and stained with silver nitrate. The apparent molecular masses of the proteins were estimated by comparison with a mixture of molecular protein markers (10–75 kDa).

### 2.4 HPLC purification of proteins and peptides

The extract of 50 pairs of salivary glands (about 50 µg) from Kashan, Isfahan and Varamin was dissolved in PBS (500 μl) separately and purification was performed using an HPLC instrument (Knauer‐Germany).

Prepared extract (50 µg in 500 μl PBS) was manually injected into C18 column (250 × 4.6 mm, TSKGel ODS‐100V 5 µm) and eluted in a linear gradient of 0.05% TFA in water (solution A) and acetonitrile containing 0.05% TFA (solution B) at a flow rate of 0.3 ml/min. In order to isolate the proteins, the column was eluted by a linear gradient of solution B from 0% to 60% for 45 min at 0.3 ml/min. The eluted peaks were monitored at 280 nm and collected manually (Belkaid et al., 2000; Boulanger et al., 2004; Ribeiro, 1992). The isolated fractions were lyophilized by a freeze dryer (Christ, Alpha 1‐2 LD plus–Germany). The purity of the isolated proteins was subsequently checked by SDS‐PAGE.

## 3 RESULTS

### 3.1 Sandflies collection

Three populations of *P. papatasi* were trapped from Isfahan province (Isfahan and Kashan locations) and Tehran province (Varamin location) in 2017–2018 (Figure 1). Males and females of different sandfly species were caught. Females *P. papatasi* were identified and considered as proven vector of zoonotic cutaneous leishmaniasis for protein extraction and analysis. A total of 2,000 female *P. papatasi* were collected during period of adult sandfly activity season in 2017 and 2018 (500 from Isfahan, 1,000 from Kashan and 500 from Varamin).

### 3.2 Protein extraction process and determination of protein profiles

**FIGURE 2:**
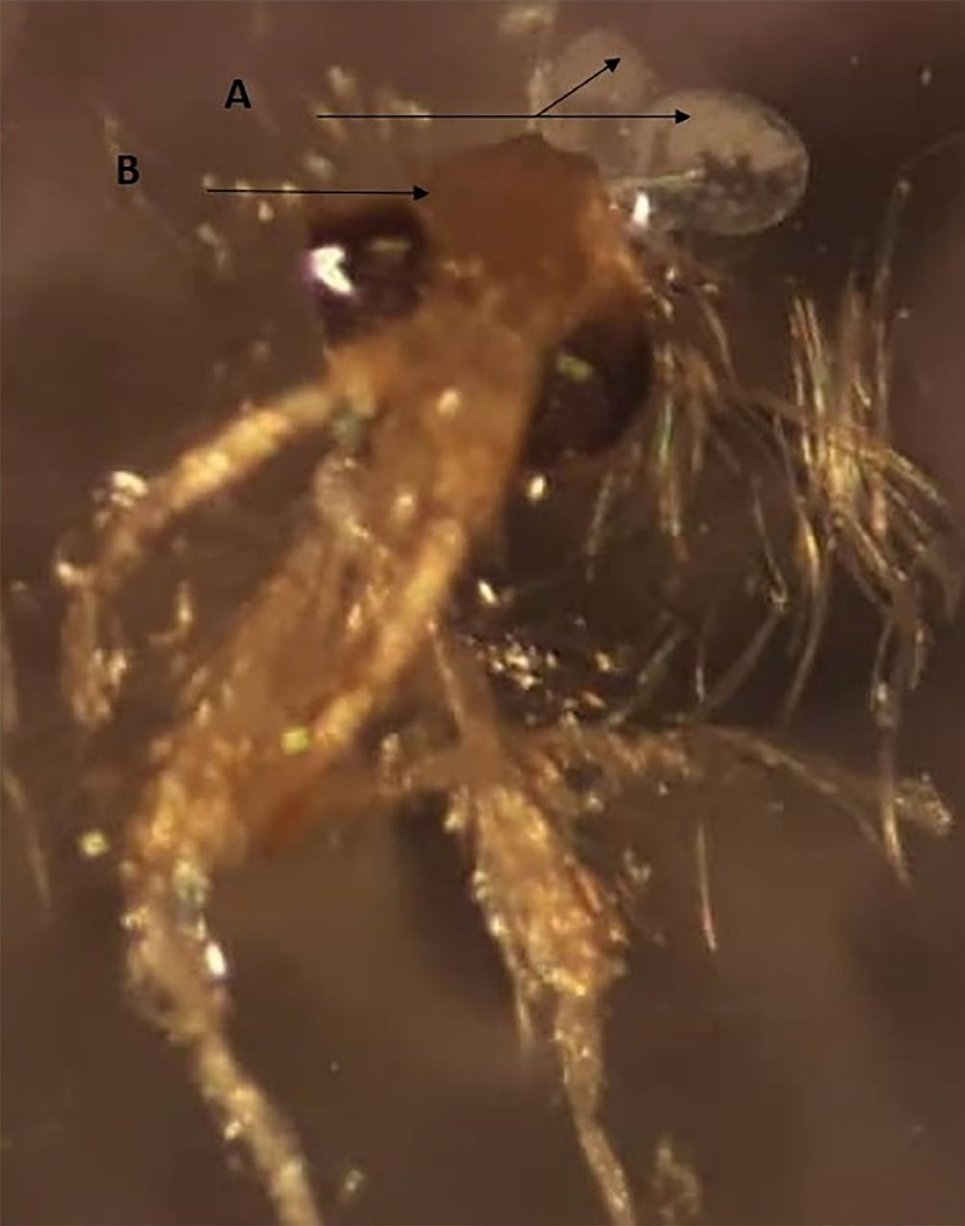
Salivary glands of *Phlebotomus papatasi* were exploited for protein extraction using stereo microscope (Nikon SMZ645) at 5× magnification and a digital camera (Canon G12 – Japan). (A) Salivary glands; (B) Head of sandfly

After protein extraction, the equivalent concentration of 50 pairs of whole salivary glands was considered approximately 1.6 µg/µl from Isfahan and 1 µg/µl from both of Varamin and Kashan (Figure 2).

**FIGURE 3:**
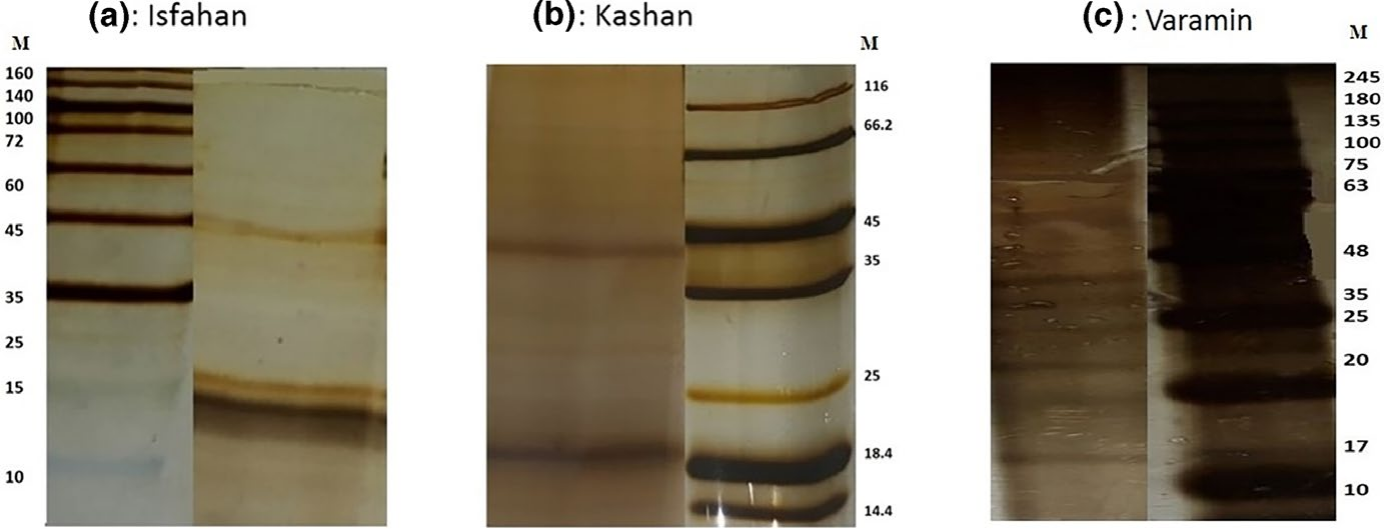
Electrophoretic profiles of whole extracted proteins. Analysed by SDS‐PAGE using 15% polyacrylamide gel and stained with silver nitrate. M: Molecular weight marker. Extracted proteins: (a) Isfahan, (b) Kashan, (c) Varamin

SDS‐PAGE showed separated bands with their molecular weights. These profiles revealed 10 proteins or peptides. Minor and major protein bands observed between 10 and 63 kDa. There were significant differences between extracted protein profiles from different ZCL foci (Figure 3).

### 3.3 Purification of PpSP15‐like proteins

**FIGURE 4:**
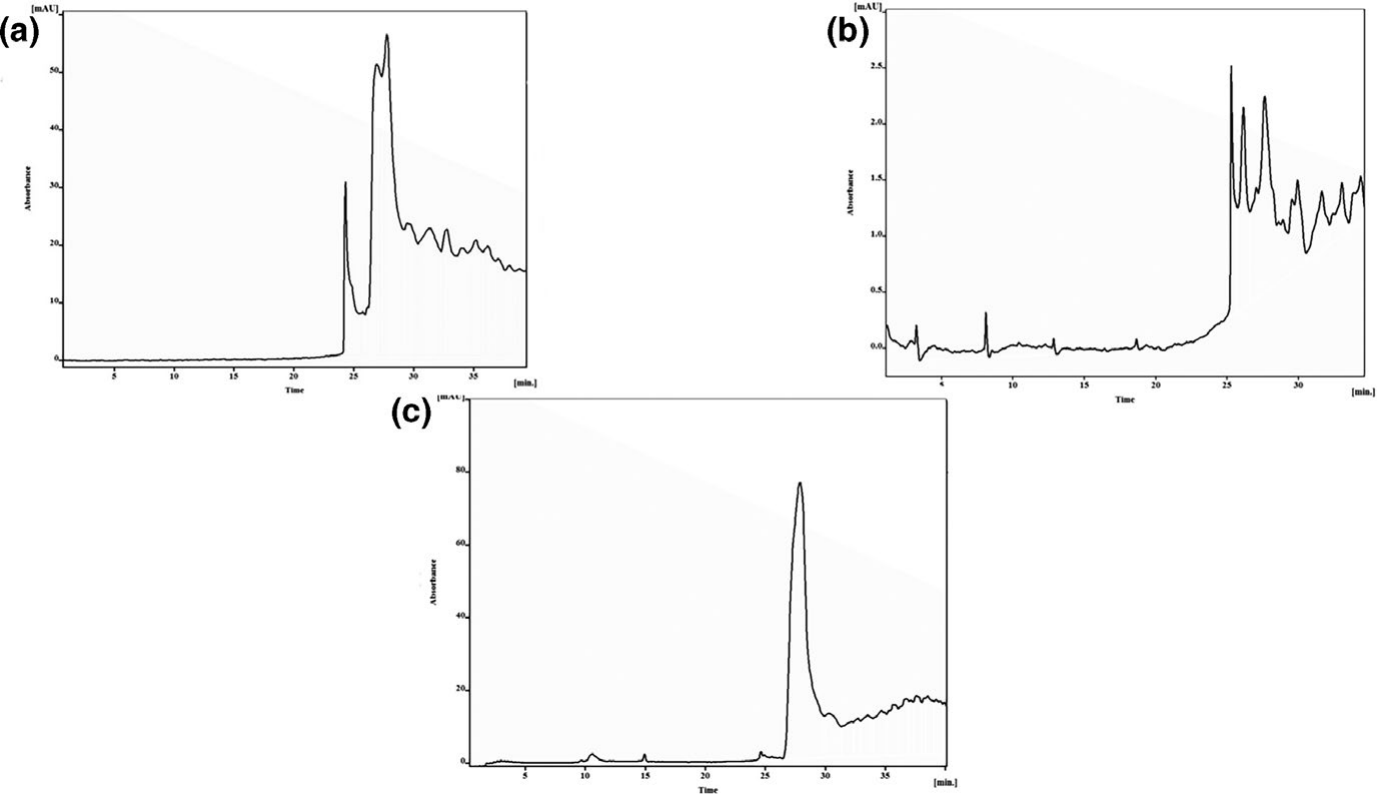
HPLC chromatograms of salivary gland proteins of *Phlebotomus papatasi* were revealed 15 peaks in 45 min. (a) Isfahan, (b) Kashan, (c) Varamin

Ten fractions of salivary gland proteins were yielded using RP‐HPLC on C18 column with a gradient protocol. Chromatograms of salivary gland proteins of *P. papatasi* were revealed 15 peaks in 45 min. The maximum optical density of the fractions was about 80 mAu for Kashan and Isfahan samples, whereas for Varamin was 5 mAu (Figure 4).

**FIGURE 5:**
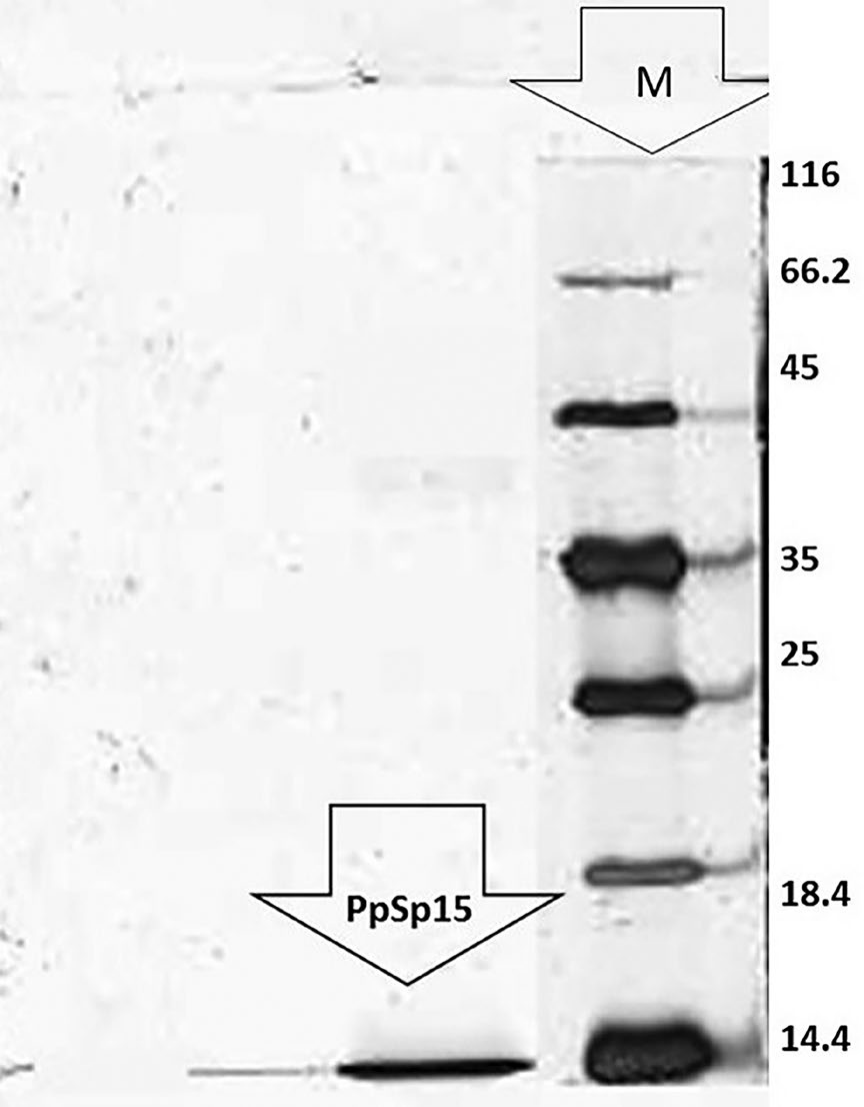
Electrophoretic profile of PpSP15. Analysed by SDS‐PAGE using 15% polyacrylamide gel and stained with silver nitrate. M: Molecular weight marker (116‐14.4 kDa)

SDS‐PAGE of all collected fractions illustrated that isolated fraction presented in 29.4 min (Isfahan), 26 to 30 min (Kashan) and 25 min (Varamin) was 15 kDa including PpSP15‐like proteins (Figure 5).

## 4 DISCUSSION

Various sandfly species introduce SP15‐like proteins as abundant proteins in salivary glands content which were described as extremely divergent proteins (Table 1). Variable SP15‐like proteins reflected among sandflies (Anderson et al., 2006; Hostomská et al., 2009), likely occurring in multiple gene copies (Elnaiem et al., 2005; Rohoušová, Subrahmanyam, et al., 2012).

**TABLE 1:** Comparison of SP15‐like proteins originated from *Phlebotomus* species

SP15 code no.	Sandflies				Best match to NCBI		MW	pI	Protein Length (aa)	Reference
	Reared in	Origin	Species	Acc. No.	Acc. No.	Species source				
PPTSP15	Tunisia	Tunisia	*P. papatasi*	JQ988879	AAL11047	*P. papatasi*	14.502	9.39	142	Abdeladhim et al. (2012)
PPTSP14.5	Tunisia	Tunisia	*P. papatasi*	JQ988878	ABI20182.1	*P. duboscqi*	14.542	9.39	142	Abdeladhim et al. (2012)
PPTSP14	Tunisia	Tunisia	*P. papatasi*	JQ988880	AAL11046	*P. papatasi*	14.736	8.85	142	Abdeladhim et al. (2012)
PabSP2	Prague	Occupied lands	*P. arabicus*	FJ538111	AAX56359.1	*P. ariasi*	14.2	9.1	138	Hostomská et al. (2009)
PabSP45	Prague	Occupied lands	*P. arabicus*	FJ538112	AAX55748.1	*P. ariasi*	14.2	9.33	137	Hostomská et al. (2009)
PabSP93	Prague	Occupied lands	*P. arabicus*	FJ538113	AAX55750.1	*P. ariasi*	14.1	9.22	139	Hostomská et al. (2009)
SP02	Spain	India	*P. argentipes*	ND	ABA12134	*P. argentipes*	15.333	10.40	139	Martin‐Martin et al. (2013)
SP01	Spain	India	*P. argentipes*	ND	ABA12133	*P. argentipes*	16.666	6.39	143	Martin‐Martin et al. (2013)
SP07	Spain	India	*P. argentipes*	ND	ABA12139	*P. argentipes*	17.333	9.78	138	Martin‐Martin et al. (2013)
ParSP03	France	—	*P. ariasi*	AY845195	AF132517.1	*L. longipalpis*	14.3	8.63	139	Oliveira et al. (2006)
ParSP06	France	—	*P. ariasi*	AY861654	AF132517.1	*L. longipalpis*	14.26	9.44	139	Oliveira et al. (2006)
ParSP08	France	—	*P. ariasi*	AY861656	AF335485.1	*P. papatasi*	14.09	8.8	140	Oliveira et al. (2006)
PpSP15	USA	Occupied lands	*P. papatasi*	HM470100.1	AAL11047	*P. papatasi*	ND	ND	142	Ramalho‐Ortigão et al. (2015)
PsSP15	Turkey	Czech	*P. papatasi*	HM560868	AAL11047	*P. papatasi*	14.7	9.07	122	Rohoušová, Subrahmanyam, et al. (2012); Rohoušová, Volfová, et al. (2012)
PorMSP12	Czech	Ethiopia	*P. orientalis*	KC170964	ADJ54084	*P. tobbi*	14.9	8.77	141	Vlkova et al. (2014)
PorMSP75	Czech	Ethiopia	*P. orientalis*	KC170975	ADJ54085	*P. tobbi*	14.7	7.99	140	Vlkova et al. (2014)
PorMSP90	Czech	Ethiopia	*P. orientalis*	KC170977	ADJ54088	*P. tobbi*	14.32	8.73	139	Vlkova et al. (2014)
PorMSP96	Czech	Ethiopia	*P. orientalis*	KC170978	ADJ54089	*P. tobbi*	14.5	8.88	141	Vlkova et al. (2014)
PorASP28	Czech	Ethiopia	*P. orientalis*	KC170938	ADJ54089	*P. tobbi*	14.53	8.88	141	Vlkova et al. (2014)
PorASP31	Czech	Ethiopia	*P. orientalis*	KC170939	ADJ54088	*P. tobbi*	14.32	8.73	138	Vlkova et al. (2014)
PorASP37	Czech	Ethiopia	*P. orientalis*	KC170940	ADJ54084	*P. tobbi*	14.91	8.77	141	Vlkova et al. (2014)
PorASP64	Czech	Ethiopia	*P. orientalis*	KC170945	ADJ54085	*P. tobbi*	14.7	7.99	140	Vlkova et al. (2014)

Abbreviations: aa, number of amino acid residues; Acc. No, accession number; MW, predicted molecular weight; ND, not done; pI, predicted isoelectric point.

Correlation between vertebrate host immune response and salivary glands proteins was defined. These proteins are recommending as a potential antigen for vaccine production against *Leishmania* infection because they can cause host immune response (Valenzuela et al., 2004). Salivary glands of *P. papatasi* with five members of small odorant‐binding protein (OBP) family contain SP12, SP14, SP14.2, SP14.5 and SP15‐like proteins (Abdeladhim et al., 2012). Molecular weight of these proteins has been predicted from 13.9 to 14.9 kDa and isoelectric point 8.0 to 9.2 (Vlkova et al., 2014).

PpSP15‐like proteins belong to OBP family, but so far the exact function of these proteins remains unknown in sandflies. However, SP15 protein (accession number: AAL11047) was shown to elicit specific humoral and cellular immunity. These caused the protection in immunized mice against *L. major* infection (Oliveira et al., 2008; Valenzuela et al., 2001).

In addition, a delayed type hypersensitivity (DTH) reaction was also observed in immunized mice inoculated of DNA plasmid coding for SP15‐like salivary protein in *P. ariasi* (accession number: AAX56359; Oliveira et al., 2006). The pre‐exposure of salivary gland proteins from colonized *P. papatasi* do not confer protection against *Leishmania* infection, whereas *L. major* were co‐inoculated with wild‐caught *P. papatasi*. These findings reveal that saliva‐based vaccine derived from colonized sandflies, affected on immune response after natural exposure and show unpredictable signs (Ben Hadji Ahmed et al., 2010). OBP family of proteins was considered for investigation from Iranian sources as endemic foci of ZCL. Our objective was to extract and compare of these orthologues proteins from different sandfly populations. This finding is novel in Iranian wild female *P. papatasi* and interested for controlling ZCL in Iran and elsewhere in the world.

In general, collecting wild sandflies from different ZCL foci is important for protein extraction and analysis. However, caching sandflies sometimes with long distances spends more time and high cost of budget. A lot of sandflies were collected and transported to the laboratory but after identifying and separating, different genera and species were found. Several times, collecting was tried until suitable numbers of female *P. papatasi* were separated and identified. Moreover, average extracted protein content per a pair of glands in each *P. papatasi* was a little and is not enough so much more samples were required (Hosseini‐Vasoukolaei, Mahmoudi, et al., 2016). Comparison between HPLC chromatograms of extracted proteomes from occupied lands, Palestine specimens as standards and our specimens from different regions revealed similarity in four major peaks from all regions except Kashan that exposed some differences (Figure 4; Belkaid et al., 2000).

There were some differences between Isfahan and Kashan chromatogram profiles and similarity in proteome analysis between Varamin and standard specimens (Belkaid et al., 2000). Moreover, evaluation of SDS‐PAGE showed distinct differences among all samples from different locations (Figure 3). There were some varieties in number and weight of specified peptides from separate foci of ZCL (Geraci et al., 2014). Some differences in number and weight of protein bands were found among Iranian specimens and the world. Losing some proteins and peptides in different ecologic and physiologic conditions could cause different protein composition of salivary glands (Abdel‐Badeia et al., 2012; Ben Hadji Ahmed et al., 2010; Geraci et al., 2014; Hosseini‐Vasoukolaei, Mahmoudi, et al., 2016; Rohoušová, Volfová, et al., 2012; Valenzuela et al., 2001).

Number variability of bands between extracted proteins from different ZCL foci was found on SDS‐PAGE. Significant differences in HPLC chromatograms were revealed in number and retention times of peaks. All conditions were the same for all collected specimens in all the time of examinations. So dissimilarities in HPLC chromatograms and SDS‐PAGE could be because of difference in protein contents which is very important in stimulating of immune system.

Some differences in protein profiles of salivary glands of *P. papatasi* from separate niches were demonstrated. One of the main hypothesis underlying these findings could be effect of these variations on immunities by molecules as antigens in vaccine development process. In the other hand, PpSP15 extracted from different populations may have dissimilar properties which have to be intended in vaccine development. The protein sequencing of Iranian foci remains to be investigated and blast to Protein Data Bank (PDB). Accurate identification of PpSP15 by LC‐MS/MS has to done in future in another research project.

## 5 CONCLUSION

Now we can conclude that investigation on salivary gland proteins of natural wild specimens from local and endemic ZCL foci should be intended separately for each area, in order to vaccine production. To innovate vector‐based and novel pharmacoactive proteins, study on salivary gland proteins of natural wild specimens is essential.

## CONFLICT OF INTEREST

The authors have no conflict of interest to declare.

## AUTHOR CONTRIBUTION

**Seyedeh Maryam Ghafari:** Conceptualization; Data curation; Formal analysis; Investigation; Methodology; Software; Visualization; Writing‐original draft. **Mahmoud Nateghi Rostami:** Data curation; Investigation; Methodology.
